# Convert Probation into Pupilage: A Golden Opportunity

**Published:** 1919-08-23

**Authors:** 


### MINISTERS OF HEALTH.

### III. Convert Probation into Pupilage: A Golden Opportunity.

Pursuing my remarks in connection with the special training of hospital pupils or probationers for work under the Health Ministry, I propose to summarise briefly the actual facts of the situation.

(i) The Ministry is anxious to secure the services of thoroughly efficient Visitors acquainted with any or all of the following: Maternity and Child Welfare, School Medical Service, Sanitary Inspection. Efficiency at such work requires a knowledge of hygiene, cookery, elementary economics, and social science. It is freely admitted by the Ministry that trained nurses and midwives are the best candidates, but it is also emphasised that their training is inadequate for the duties involved.

(ii) Present occupants of appointments as Health Visitors will not be disturbed, but in future none will be appointed except trained candidates, and grants under Maternity and Child Welfare Regulations will be restricted to such.

(iii) Training must be in connection with a recognised institution, preferably a university institution. "Full" courses, extending over two years, are laid down as the minimum for ordinary students, and "shortened" courses, extending over one year for "trained nurses, and other persons already possessing substantial knowledge or experience." Ordinary students may begin at eighteen, and "they must have received a good previous education, preferably a secondary-school education." An examination must be passed after training, and the following are regarded as the essential subjects:

- Elementary physiology.
- Methods of artisan cooking and household management.
- Hygiene.
- Infectious and communicable diseases.
- Maternity, infant and child welfare.
- Elementary economics and social problems.

The importance of practical work during training is accentuated.

### Situation Fluid.

At present the situation is in a fluid state. As the regulations have it—"at the present time it is neither practicable nor desirable to indicate in anything more than broad outline the subjects to be included in the course. . . . It is recognised that all proposals will be experimental to begin with, and that some considerable variation may reasonably be allowed.

### To Be or Not to Be.

The question at issue is, will nurses' training-schools adapt themselves to this programme? It is, obviously, a great opportunity for a drastic reform of the probationary system, an opportunity which offers the immediate possibility of making nursing in the full sense a profession. Much has been said in the past about a one-portal system, which may be quite right as setting up a minimum standard, but wholly retrogressive if intended to signify complete uniformity of ultimate achievement. There is no such arrangement as this in any profession or in any university, and my personal opinion of the "one-portal" expression is that it is a catchword designed to appeal to the semi-trained, and to belittle the highly trained. The Local Government Board, before it was transformed into the Ministry of Health, recognised nurses as trained if they had spent three years in a Poor Law institution, where there was no operating-theatre and none of the equipment of a modern hospital. This may suffice as qualification for the description "R.N.," but it is very far from sufficient to give nursing a professional status. The Board of Education and the Ministry of Health are altogether outside the region of the factions which distinguish the nursing world. They are Departments of State which are bound to keep in touch with the progress of the world in civilisation and education. The Ministry of Health needs efficient workers, and they do not conceal their preference for nurses. But it is made quite clear that in the first place they must be women of education, and in the second that they must have extended professional training. Referring to Health Visitors and their outlook, the *Times* says that "the steady rise of the salary of trained certificated teachers is some indication of the remuneration that will probably be given for such work, as the Visitors are likely to be treated on the same basis; and there will be a very promising career under the larger authorities for those women who have superior organising capacity and grasp of affairs."

### Grants for Training.

The Ministry of Health undertakes to make grants to suitable training institutions. These are undefined, but courses must, "as a rule," be conducted by or in close association with a university institution. The sum of £40 is payable for each student who takes a full course, and £20 for a shortened course. Surely this whole situation is one calculated to test the enterprise and the capacity of the College of Nursing. It ought not to be without the bounds of possibility to establish such an institution as that prescribed by the new regulations as an integral part of the College of Nursing, and under the ægis of the University of London. Such a school would be almost, if not altogether, self-supporting, and would go further towards the uplifting of nurses than anything yet that has been done on their behalf. But the College will obviously be helpless unless some of the larger of the Metropolitan hospitals co-operate by converting probation into pupilage, reducing the hours, and providing facilities for study.

The opportunity is a golden one, and if not availed of without delay may be lost. Other institutions are already becoming busy, and the regulations provide that "grants will first be payable for the session commencing August 1, 1919."

IERNE.

